# Association between low-carbohydrate diet score and childhood obesity: a national population-based study

**DOI:** 10.1186/s12887-026-06835-1

**Published:** 2026-04-11

**Authors:** Fei Wang, Zhen Liu, Fengfeng Liu, Xue Qiao, Zhuhui Zhao, Lan Zhang

**Affiliations:** 1 Department of Pediatrics, The Fifth People’s Hospital of Shanghai, Fudan University, 128 Ruili Road, Shanghai, 200240 China; 2 Department of Pediatric Endocrinology and Inherited Metabolic Diseases, Children’s Hospital of Fudan University, Shanghai, 201102 China

**Keywords:** Childhood obesity, Low-carbohydrate diets, NHANES, Cross-sectional study

## Abstract

**Background:**

Aimed to investigate the association between a low-carbohydrate diets (LCD) score and the prevalence of obesity in a large, nationally representative sample of children and adolescents in the United States.

**Methods:**

This cross-sectional study utilized data from participants aged 2–17 years from the National Health and Nutrition Examination Survey (NHANES) spanning 2003 to 2018. The exposure of interest was an LCD score, calculated based on the relative intake of carbohydrates, proteins, and fats from the mean of two 24-hour dietary recalls. The primary outcome was obesity, defined as a body mass index at or above the 95th percentile for age and sex. Multivariable logistic regression analysis with three progressive models, restricted cubic spline (RCS) analysis, and subgroup analyses were performed.

**Results:**

A total of 12,684 participants were included in the final analysis, including 7,392 with obesity and 11,679 without obesity. After comprehensive adjustment for demographic, socioeconomic, and dietary covariates (including total energy intake), a higher LCD score was positively associated with the odds of obesity. When treated as a continuous variable, each one-unit increase in the LCD score corresponded to a 2% increase in the odds of obesity (OR = 1.02, 95% CI: 1.01–1.03, *P* < 0.001). When categorized into quartiles, participants in the highest quartile (Q4) had 40% higher odds of obesity compared to those in the lowest quartile (Q1) (OR = 1.40, 95% CI: 1.17–1.68, *P* < 0.001). The RCS analysis confirmed a significant non-linear association between the LCD score and the odds of obesity (P for non-linearity = 0.001). Furthermore, subgroup analyses indicated that the association varied across strata; for instance, a significant positive association was observed in children aged 12 years and younger but not in older adolescents, and was stronger in males than in females, though formal tests for interaction were not statistically significant.

**Conclusion:**

In this nationally representative sample of U.S. children and adolescents, a higher LCD score was associated with increased odds of obesity. This finding suggests that a dietary pattern characterized by a lower proportion of carbohydrates and higher proportions of fat and protein may reflect poor overall dietary quality rather than a structured, health-promoting diet, which is associated with higher obesity prevalence.

**Clinical trial number:**

Not applicable.

**Supplementary Information:**

The online version contains supplementary material available at 10.1186/s12887-026-06835-1.

## Background

The escalating prevalence of childhood obesity has become one of the most formidable public health challenges of the 21st century [1]. Globally, rates have risen dramatically over the past several decades, affecting not only high-income nations but also an increasing number of low- and middle-income countries [2, 3]. This epidemic carries profound and enduring health consequences. Obesity in childhood is a strong predictor of adult obesity and is associated with a premature onset of a range of noncommunicable diseases, including type 2 diabetes, cardiovascular diseases such as hypertension and dyslipidemia, non-alcoholic fatty liver disease, and certain types of cancer [4, 5]. This highlights the urgent need to investigate key modifiable lifestyle factors, particularly the role that specific and popular dietary patterns play in the pediatric population.

In the broader nutritional landscape, low-carbohydrate diets (LCDs) have gained considerable popularity as a strategy for weight management and metabolic health improvement, primarily within adult populations. A substantial body of evidence from clinical trials and meta-analyses suggests that LCDs can be effective for short-term weight loss and can favorably alter cardiometabolic risk factors, such as improving triglyceride and HDL cholesterol levels [6]. These diets typically involve reducing the intake of carbohydrates while increasing the proportional intake of protein and fat [7, 8]. The success of these dietary patterns in adults has prompted questions about their potential applicability and safety in other populations, including children and adolescents.

However, the pediatric population presents a unique physiological context that complicates the direct translation of adult dietary strategies. Childhood and adolescence are critical periods of rapid growth and development, with distinct and non-negotiable nutritional requirements. Carbohydrates serve as the primary energy source for the developing brain and for physical activity, proteins are essential for building and repairing tissues, and healthy fats are vital for neurological development and hormone production [9]. Consequently, restrictive dietary patterns, including some forms of LCDs, pose potential risks. These risks include inadequate intake of essential micronutrients and fiber, potential impairment of growth, and the development of disordered eating behaviors, which can have lasting physical and psychological consequences [10]. This creates a fundamental tension between the need to address rising obesity rates and the imperative to ensure adequate nutrition for healthy development.

Despite the widespread discussion surrounding LCDs, there remains a significant gap in the scientific literature regarding their association with obesity in the general pediatric population. While some small-scale clinical trials have explored the use of therapeutic LCDs for weight management in children with obesity, large-scale, population-based studies examining the dietary patterns of children as they exist in the real world are scarce [11, 12]. It is unknown how a dietary pattern that is naturally lower in carbohydrates and higher in fat and protein, as captured by a dietary score, relates to obesity risk in a nationally representative sample. Therefore, this cross-sectional study aimed to fill this critical knowledge gap by investigating the association between a LCD score and the prevalence of obesity among children and adolescents, utilizing extensive data from the National Health and Nutrition Examination Survey (NHANES).

## Methods

### Study design and participants

This study utilized a retrospective cross-sectional design to analyze data from NHANES, conducted from 2003 to 2004 to 2017–2018. NHANES is a nationally representative survey that assesses the health and nutritional status of the U.S. civilian non-institutionalized population using a complex, multistage, stratified probability sampling design [13]. All analyses in this study incorporated the appropriate NHANES survey weights to account for this complex sampling design, non-response, and oversampling of certain subgroups, thereby producing nationally representative estimates. The study protocol was approved by the National Center for Health Statistics Research Ethics Review Board, and written informed consent was obtained from all participants or their legal guardians.

Participants for this analysis were selected from the eight combined NHANES cycles (2003–2018). The inclusion criterion was an age between 2 and 17 years [14], representing the standard pediatric age range for which CDC BMI percentiles are validated. Exclusion criteria were applied sequentially: participants aged less than 2 years or greater than 17 years were excluded, followed by those with missing Body Mass Index (BMI) data. Subsequently, participants who lacked complete dietary intake data from two 24-hour recalls were also excluded. Due to a high proportion of missing data (> 30%), covariates such as physical activity, sleep duration, and sedentary time were not included (Table S2). Participants with missing information on the remaining selected covariates were excluded (complete-case analysis), resulting in a final analytical sample of 12,684 participants.

### Definition of variables

The primary exposure variable was the LCD score [15]. This score was constructed to reflect the relative proportions of macronutrients in the diet. It was calculated by summing the points assigned for the percentage of energy derived from carbohydrates, protein, and fat based on the mean of two 24-hour dietary recalls. While this scoring system assigns equal weights to each macronutrient, this approach facilitates cross-study comparability. For each macronutrient, points from 0 to 10 were awarded based on predefined categories of relative intake. A higher score for carbohydrates was given for lower intake, while higher scores for protein and fat were given for higher intakes. The total LCD score ranged from 0 to 30, with a higher score indicating a diet compositionally lower in carbohydrates and higher in protein and fat. The scores were categorized into four non-overlapping quartiles: Q1 (≤ 10), Q2 (11–16), Q3 (17–22), and Q4 (≥ 23). The specific criteria for assigning points for each macronutrient are detailed in Table S1.

The primary outcome variable was childhood obesity. This was determined using BMI, calculated as weight in kilograms divided by the square of height in meters (kg/m2). The calculated BMI values were then converted to age- and sex-specific BMI percentiles based on the 2000 Centers for Disease Control and Prevention (CDC) Growth Charts [16, 17]. In accordance with established definitions, obesity was defined as a BMI at or above the 95th percentile for age and sex, a standard cut-off widely used to identify pediatric populations at high risk for metabolic complications.

Covariates were selected based on their established associations with childhood obesity and dietary patterns. These included demographic and socioeconomic variables. Demographic variables were age (continuous, in years), sex (male or female), and race/ethnicity (categorized as Mexican American, Other Hispanic, Non-Hispanic White, Non-Hispanic Black, or Other Race), child education level, and parental education level. The primary socioeconomic variable was the Poverty Income Ratio (PIR), which is the ratio of family income to the poverty threshold. For this analysis, PIR was dichotomized into two categories: household income below the poverty line (PIR < 1.0) and household income at or above the poverty line (PIR ≥ 1.0), based on the data structure provided. Total daily energy intake (in Kcal), derived from the 24-hour dietary recalls, was also included as a dietary covariate.

### Statistical analysis

All statistical analyses were conducted using R software (version 4.2.0) and accounted for the complex survey design of the NHANES by applying the appropriate sample weights (WTDR2D/8), strata (SDMVSTRA), and primary sampling units (SDMVPSU). Baseline characteristics of the study population were summarized and stratified by obesity status. Continuous variables were presented as mean ± standard deviation (SD) and compared between groups using the weighted Wilcoxon rank-sum test. Categorical variables were presented as weighted percentages and compared using the weighted chi-square test.

Multivariable logistic regression was used to examine the association between the LCD score and the odds of childhood obesity, calculating odds ratios (ORs) and 95% confidence intervals (CIs). The LCD score was analyzed both as a continuous variable (per 1-unit increase) and as a categorical variable based on quartiles (Q1-Q4), with the lowest quartile (Q1) serving as the reference group. Four hierarchical models were constructed: Model 1 was unadjusted; Model 2 was adjusted for age and sex; and Model 3, the fully adjusted model, was adjusted for age, sex, race/ethnicity, and PIR; and Model 4, the fully adjusted model, further adjusted for total energy intake, education level, and parental education level.

To explore the potential for a non-linear dose-response relationship between the continuous LCD score and the odds of obesity, a restricted cubic spline (RCS) analysis was performed [18]. The RCS model, with four knots placed at the 5th, 35th, 65th, and 95th percentiles of the LCD score distribution, was fitted within the fully adjusted logistic regression framework.

Subgroup analyses were conducted to evaluate the consistency of the association between the continuous LCD score and obesity across different strata of age, sex, race/ethnicity, and PIR. A two-sided p-value less than 0.05 was considered statistically significant for all descriptive analyses. For the fully adjusted model, multicollinearity was assessed using the Variance Inflation Factor (VIF), which indicated no severe collinearity (Table S3). Model calibration and discrimination were evaluated using the F-adjusted Hosmer-Lemeshow test and the Area Under the Curve (AUC), respectively. Two sensitivity analyses were conducted: one excluding participants with implausible energy intakes (< 500 or > 5000 Kcal), and another mutually adjusting for individual macronutrient percentages.

## Results

### Baseline characteristics

**Fig. 1:**
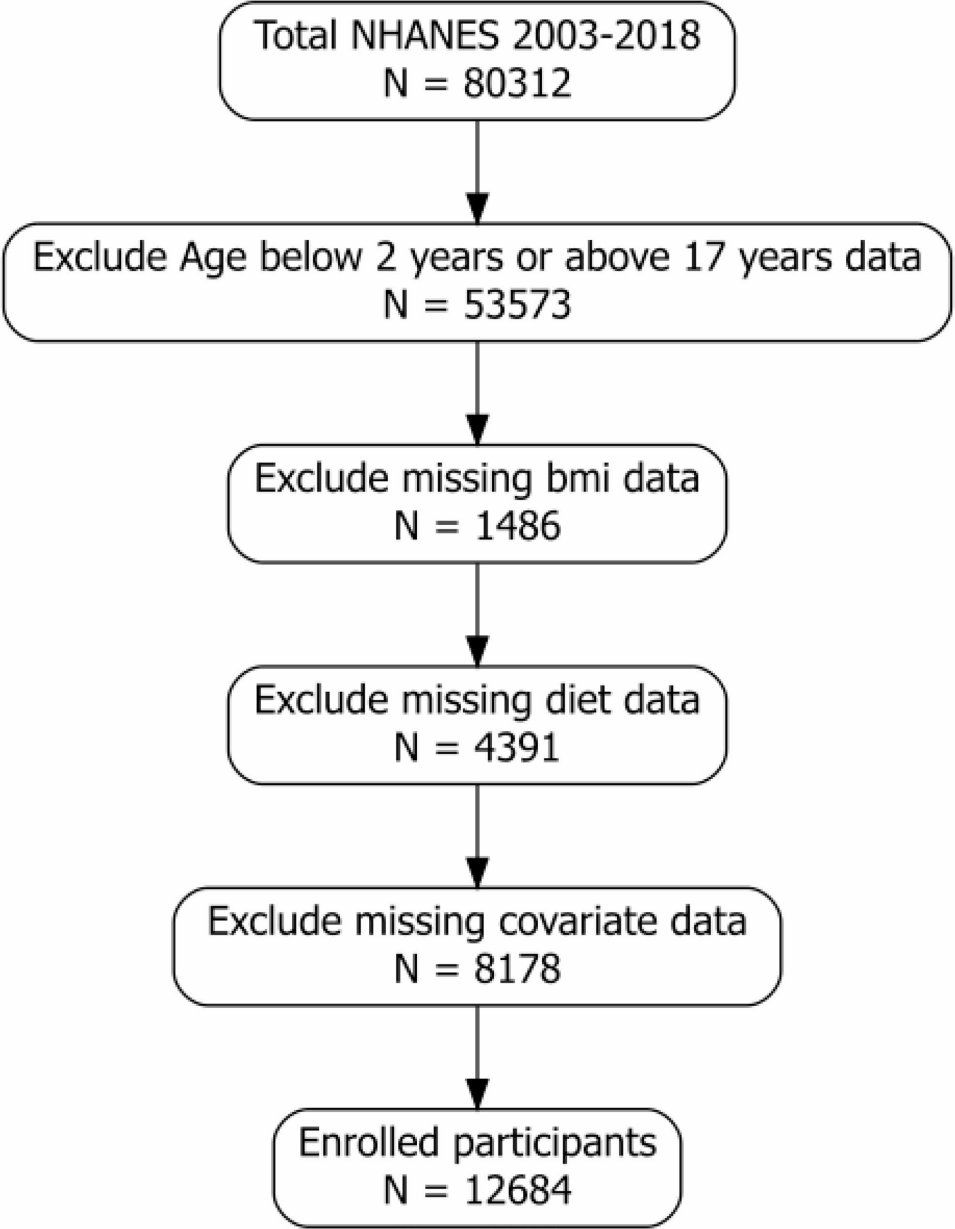
Chart depicting the selection criteria used for this study

The participant selection process is detailed in Fig. 1. From an initial pool of 80,312 participants in the NHANES cycles, we excluded 53,573 individuals for being outside the target age range, 1,486 for missing BMI data, 4,391 for incomplete dietary data, and 8,178 for missing covariate information. This resulted in a final study cohort of 12,684 children and adolescents, representing a weighted population of 39,591,987 U.S. youth aged 2–17 years. The baseline demographic, anthropometric, and dietary characteristics of these participants, stratified by LCD score quartiles, are presented in Table 1.

**Table 1:** Weighted basic characteristics

Variables	Overall (*N* = 39591987)	Q1 (*N* = 10113971)	Q2 (*N* = 10214760)	Q3 (*N*-10368477)	Q4 (*N* = 8894780)	*P*
Age (year)	11.6 (3.5)	11.1 (3.5)	11.4 (3.4)	11.6 (3.4)	12.3 (3.4)	< 0.001
Sex, n (%)						< 0.001
Male	19976956.2 (50.5)	4592786.2 (45.4)	5084653.0 (49.8)	5269628.7 (50.8)	5029888.2 (56.5)	
Female	19615031.2 (49.5)	5521184.8 (54.6)	5130106.9 (50.2)	5098848.0 (49.2)	3864891.6 (43.5)	
Race, n (%)						0.128
Mexican American	5510773.2 (13.9)	1308110.6 (12.9)	1476581.6 (14.5)	1576888.5 (15.2)	1149192.5 (12.9)	
Other Hispanic	2335333.3 (5.9)	639803.7 (6.3)	615460.7 (6.0)	608419.6 (5.9)	471649.3 (5.3)	
Non-Hispanic White	23170274.0 (58.5)	6017915.5 (59.5)	5917880.6 (57.9)	5833776.2 (56.3)	5400701.5 (60.7)	
Non-Hispanic Black	5551573.2 (14.0)	1352448.0 (13.4)	1329515.9 (13.0)	1636508.5 (15.8)	1233100.8 (13.9)	
Other Race	3024033.8 (7.6)	795693.2 (7.9)	875321.1 (8.6)	712883.9 (6.9)	640135.7 (7.2)	
Education level						0.006
less than 9th grade	33770866.0 (85.3)	8765908.8 (86.7)	8864271.1 (86.8)	8880866.8 (85.7)	7259819.3 (81.6)	
9th grade or above	5821121.4 (14.7)	1348062.2 (13.3)	1350488.8 (13.2)	1487609.9 (14.3)	1634960.5 (18.4)	
PIR, n (%)						0.418
Household income is below the poverty line.	8585885.3 (21.7)	2204484.1 (21.8)	2263888.8 (22.2)	2333707.0 (22.5)	1783805.4 (20.1)	
Household income is above the poverty line	31006102.1 (78.3)	7909486.9 (78.2)	7950871.1 (77.8)	8034769.7 (77.5)	7110974.4 (79.9)	
Obesity						0.001
No	23601283.3 (59.6)	6232513.7 (61.6)	6444771.2 (63.1)	5773064.1 (55.7)	5150934.3 (57.9)	
Yes	15990704.1 (40.4)	3881457.3 (38.4)	3769988.7 (36.9)	4595412.6 (44.3)	3743845.5 (42.1)	
Parental education level						0.283
Below high school	7486071.7 (18.9)	1984961.7 (19.6)	1852623.8 (18.1)	2096872.8 (20.2)	1551613.5 (17.4)	
High school or above	32105915.7 (81.1)	8129009.3 (80.4)	8362136.1 (81.9)	8271603.9 (79.8)	7343166.3 (82.6)	
Age (mean (SD))	11.6 (3.5)	11.1 (3.5)	11.4 (3.4)	11.6 (3.4)	12.3 (3.4)	< 0.001
Weight (Kg)	49.6 (21.7)	46.9 (21.8)	47.8 (20.7)	50.4 (21.9)	53.6 (21.8)	< 0.001
Height (CM)	150.2 (18.7)	147.1 (19.0)	149.2 (18.5)	150.5 (18.5)	154.4 (18.1)	< 0.001
BMI (Kg/m^2^)	20.9 (5.5)	20.5 (5.6)	20.5 (5.2)	21.2 (5.6)	21.6 (5.6)	< 0.001
Waist-to-Height Ratio	73.0 (15.4)	71.5 (15.8)	71.9 (14.9)	73.7 (15.5)	75.4 (15.2)	< 0.001
LCD Score	16.0 (7.5)	6.2 (3.0)	13.6 (1.7)	19.4 (1.7)	25.8 (2.2)	< 0.001
Fat relative intake (%)	33.1 (5.9)	26.9 (4.1)	31.3 (3.2)	35.1 (3.5)	39.7 (3.7)	< 0.001
Protein relative intake (%)	14.5 (3.5)	12.6 (3.0)	14.3 (3.1)	15.0 (3.4)	16.3 (3.4)	< 0.001
Carbohydrate relative intake (%)	52.4 (6.9)	60.4 (4.2)	54.4 (2.6)	49.9 (3.2)	44.0 (4.4)	< 0.001
Total energy intake (Kcal)	2026.2 (710.3)	1674.6 (547.2)	1986.5 (665.7)	2071.6 (644.4)	2418.7 (783.5)	< 0.001

*PIR* Poverty Income Ratio, *BMI* Body Mass Index, *LCD* Low-Carbohydrate Diet

### Association of LCD score with childhood obesity

The results of the multivariable logistic regression analyses examining the association between the LCD score and childhood obesity are detailed in Table 2. In the unadjusted model (Model 1), each one-unit increase in the LCD score was associated with a 2% increase in the odds of obesity (OR = 1.02, 95% CI: 1.01–1.03). In the fully adjusted model (Model 4), which controlled for age, sex, race/ethnicity, PIR, total energy intake, education level, and parental education level, each one-unit increase in the LCD score was associated with higher odds of obesity (OR = 1.02, 95% CI: 1.01–1.03, *P* < 0.001).

**Table 2:** Multivariate logistic regression

	Model 1		Model 2		Model 3		Model 4	
	OR (95%CI)	*P*	OR (95%CI)	*P*	OR (95%CI)	*P*	OR (95%CI)	*P*
LCD score	1.01 (1.00-1.02)	0.007	1.01 (1.00-1.02)	0.013	1.01 (1.00-1.02)	0.013	1.02 (1.01–1.03)	< 0.001
Q1	ref	ref	ref	ref	ref	ref	ref	ref
Q2	0.94 (0.81–1.10)	0.421	0.93 (0.80–1.09)	0.383	0.94 (0.80–1.09)	0.392	1.01 (0.87–1.19)	0.859
Q3	1.28 (1.07–1.52)	0.007	1.27 (1.06–1.51)	0.009	1.25 (1.05–1.50)	0.015	1.39 (1.16–1.68)	< 0.001
Q4	1.17 (0.99–1.37)	0.059	1.14 (0.97–1.34)	0.103	1.15 (0.98–1.35)	0.089	1.40 (1.17–1.68)	< 0.001
*P* trend		0.002		0.005		0.006		0.006

Model 1: Unadjusted model

Model 2: Adjusted for Age, Sex

Model 3: Adjusted for Age, Sex, Race, PIR,

Model 4: Adjusted for Age, Sex, Race, PIR, Total energy intake, Education level, Parental education level

**Fig. 2:**
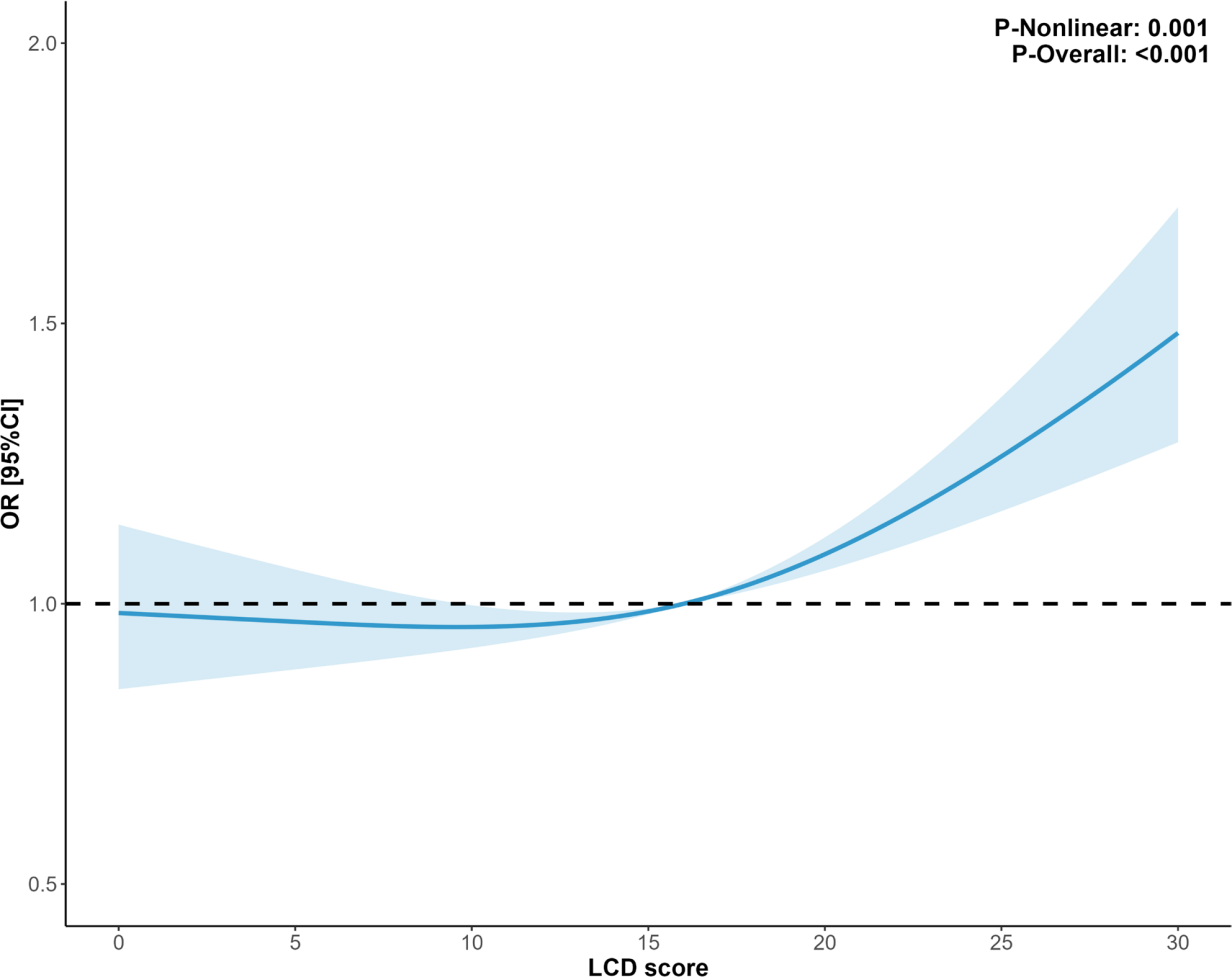
RCS curve fits the association of LCD score and childhood obesity. Adjusted for Age, Race, PIR, Sex

When the LCD score was analyzed in quartiles, a clear dose-response relationship emerged. In the fully adjusted model (Model 3), compared to participants in the lowest quartile (Q1), those in the second quartile (Q2) showed no significant difference in the odds of obesity (OR = 0.96, 95% CI: 0.84–1.08). However, participants in the third (Q3) and fourth (Q4) quartiles had significantly higher odds of obesity. The OR for Q3 was 1.39 (95% CI: 1.16–1.68, *P* < 0.001), and the OR for Q4 was 1.40 (95% CI: 1.17–1.68, *P* < 0.001). The test for trend across the quartiles was highly significant (P_trend_ = 0.006), indicating a progressive increase in the odds of obesity with increasing quartiles of the LCD score. The restricted cubic spline analysis, adjusted for all covariates, revealed a significant non-linear association between the continuous LCD score and the odds of childhood obesity (P for non-linearity = 0.001). As illustrated in Fig. 2, the odds of obesity increased with the LCD score. The relationship appeared to be steepest in the lower to middle range of the score, after which the curve began to flatten at higher LCD scores, suggesting that the increase in odds diminishes at the upper end of the score distribution.

### Subgroup analyses

**Fig. 3:**
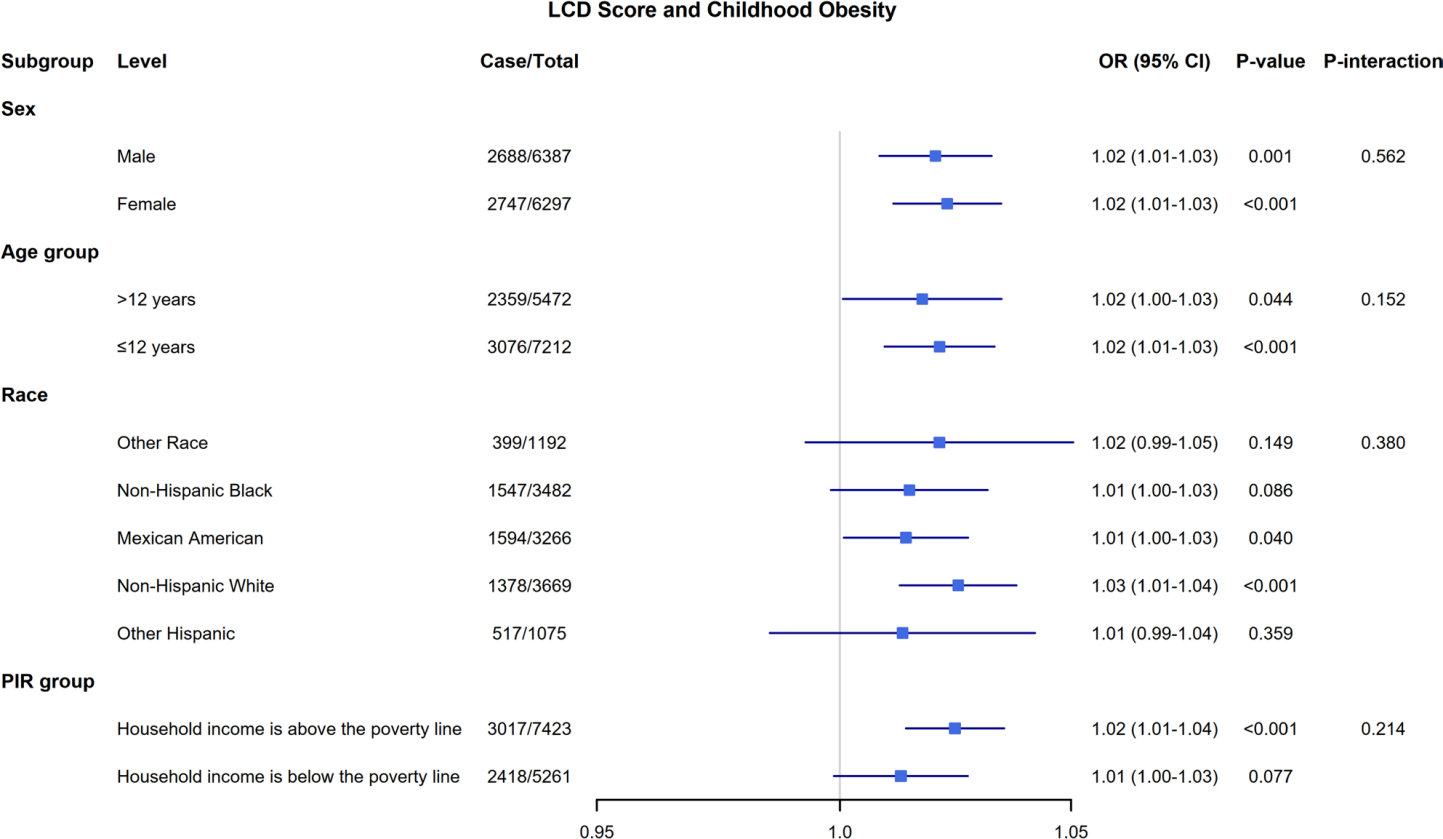
Subgroup analysis of the association between LCD score and childhood obesity

Subgroup analyses were performed, stratifying by age, sex, race/ethnicity, and PIR (Fig. 3**)**, Although significant positive associations were observed in specific strata (e.g., males, children ≤ 12 years), the formal tests for interaction were not statistically significant for age (*P*-interaction = 0.083), sex (*P*-interaction = 0.562), race/ethnicity (*P*-interaction = 0.380), or PIR (*P*-interaction = 0.214). Therefore, these subgroup patterns should be interpreted with caution. When stratified by race/ethnicity, the association was statistically significant among Non-Hispanic White (*P* < 0.001) and Mexican American participants (*P* = 0.040), but not in other groups. The positive association was present regardless of socioeconomic status, being significant in children from households above the poverty line (*P* < 0.001), but not below (*P* = 0.077) the poverty line. Stratification by sex revealed a significant association in both males (*P* = 0.001) and females (*P* < 0.001).

In the first sensitivity analysis excluding 83 participants with implausible energy intakes (< 500 or > 5000 Kcal), the association remained robust (Model 4: Q4 vs. Q1 OR = 1.40, 95% CI: 1.17–1.68, *P* < 0.001) **(**Table 3**)**. Furthermore, multivariate adjusted models for individual macronutrients indicated that higher percentages of fat (OR = 1.02, 95% CI: 1.00-1.03, *P* = 0.005) and protein (OR = 1.03, 95% CI: 1.01–1.05, *P* = 0.001) were independently associated with increased odds of obesity **(**Table 4**)**.

**Table 3:** Association between LCD score and childhood obesity after excluding participants with implausible energy intakes

	Model 1		Model 2		Model 3		Model 4	
	OR (95%CI)	*P*	OR (95%CI)	*P*	OR (95%CI)	*P*	OR (95%CI)	*P*
LCD SCORE	1.01 (1.00-1.02)	0.002	1.01 (1.00-1.02)	0.004	1.01 (1.00-1.02)	0.003	1.02 (1.01–1.03)	< 0.001
Q1	ref	ref	ref	ref	ref	ref	ref	ref
Q2	0.96 (0.82–1.12)	0.572	0.95 (0.82–1.11)	0.522	0.95 (0.82–1.11)	0.530	1.02 (0.87–1.20)	0.773
Q3	1.30 (1.09–1.55)	0.004	1.29 (1.08–1.54)	0.006	1.27 (1.06–1.52)	0.010	1.40 (1.16–1.69)	< 0.001
Q4	1.21 (1.02–1.42)	0.025	1.18 (1.00-1.39)	0.047	1.19 (1.01–1.40)	0.042	1.40 (1.17–1.68)	< 0.001
*P* trend		< 0.001		0.002		0.002		< 0.001

Model 1: Unadjusted model

Model 2: Adjusted for Age, Sex

Model 3: Adjusted for Age, Sex, Race, PIR,

Model 4: Adjusted for Age, Sex, Race, PIR, Total energy intake, Education level, Parental education level

**Table 4:** Adjusted associations of macronutrient energy percentages with childhood obesity

Nutrient	Univariate Model		Multivariate Model	
	OR (95%CI)	*P*	OR (95%CI)	*P*
carbs_percent	0.98 (0.97–0.99)	< 0.001	Reference	
fat_percent	1.02 (1.01–1.03)	0.002	1.02 (1.00–1.03)	0.005
protein_percent	1.03 (1.01–1.05)	< 0.001	1.03 (1.01–1.05)	0.001

## Discussion

In this large, nationally representative cross-sectional study of U.S. children and adolescents, a higher LCD score demonstrated a modest but statistically significant positive association with the prevalence of obesity after adjustment for key demographic and socioeconomic factors. This association was consistently observed when the score was analyzed as a continuous variable and in quartiles, and it followed a significant non-linear pattern. This finding provides an important initial insight, suggesting that a dietary pattern that is de facto lower in carbohydrates within the general pediatric population may not be protective and could instead be a marker for an overall dietary profile associated with increased odds of obesity.

The central finding of a positive association between a higher LCD score and obesity may seem counterintuitive, given the popular perception of low-carbohydrate diets as a tool for weight loss. However, a critical interpretation is that the LCD score, within the context of a general population survey, does not likely represent a structured, therapeutic, or health-conscious dietary intervention. Instead, it more plausibly serves as a proxy for a poor-quality dietary pattern. Furthermore, given the cross-sectional design, reverse causation is a strong possibility; children with obesity (or their caregivers) might actively modify their diets towards lower carbohydrate intake as a weight management strategy. Such a pattern is characterized by the substitution of nutrient-dense, fiber-rich carbohydrate sources like fruits, vegetables, and whole grains with energy-dense, often highly processed sources of fat and protein [19]. This interpretation is strongly supported by the baseline data, which showed no significant difference in total caloric intake between the obese and non-obese groups, implying that the composition and quality of the diet, rather than total energy, may underlie the observed association. This aligns with extensive literature identifying “Western” or unhealthy dietary patterns—high in processed meats, refined grains, and unhealthy fats—as being strongly associated with obesity [20–22].

When contextualized within the literature on specific macronutrients, the findings provide further mechanistic plausibility. The LCD score in this study rewards higher protein intake, a factor that has been implicated in early-life weight gain. The “early protein hypothesis” posits that excessive protein intake during critical developmental windows can accelerate growth and promote adipogenesis, which is associated with a higher risk of obesity later in childhood [23–25]. Studies have shown that high protein intake, particularly from animal sources like dairy and meat, is associated with a higher BMI in children [24]. Recent research using the same NHANES database also found that a higher dietary protein proportion was positively associated with the risk of overweight and obesity in American children [26]. While some research suggests high protein intake may be beneficial for children already managing obesity [27], our population-level findings align with concerns about excessive protein in the general pediatric diet. Similarly, the quality of dietary fat is paramount. A high LCD score could reflect a high intake of saturated and trans fats from processed foods and animal products, which are known to contribute to adverse metabolic outcomes, rather than beneficial unsaturated fats from plant sources [28].

The dietary pattern captured by the LCD score may also be closely linked to the high consumption of ultra-processed foods (UPFs) in the American pediatric diet [19]. NHANES data indicate that children and adolescents derive over 60% of their daily calories from UPFs, which include products like sweetened beverages, savory snacks, and processed meats [29, 30]. These foods are engineered to be hyper-palatable, energy-dense, and poor in essential nutrients and fiber, a profile that aligns with a high LCD score. Furthermore, the observed association between lower socioeconomic status and higher obesity prevalence in our baseline data is consistent with literature showing that lower-income households may have greater reliance on less expensive, energy-dense, and often ultra-processed foods [31, 32]. Thus, the LCD score may be capturing a complex interplay between dietary quality, food processing, and socioeconomic determinants of health.

An important and nuanced finding of this study was the significant heterogeneity observed in the subgroup analyses, suggesting the association between the LCD score and obesity is not uniform and is likely modified by key demographic factors. The association was notably stronger in younger children (≤ 12 years) and became non-significant in adolescents. This age-dependent association may be multifactorial. Younger children are in a critical period of growth where dietary quality has a profound impact, and their food choices are largely determined by their household environment. In contrast, the lack of association in adolescents could be attributed to the major metabolic and hormonal shifts of puberty, greater dietary autonomy leading to different eating patterns outside the home, and the increasing influence of unmeasured confounders such as physical activity [33]. Similarly, the association was significant in males but not in females. This could reflect sex-based differences in body composition, energy expenditure, or the specific food sources contributing to a higher LCD score. It is well-established that the drivers of obesity and the associations with dietary patterns can differ significantly between sexes during childhood and adolescence, a complexity often discussed in the context of overall pediatric obesity management [2]. These findings underscore that the relationship between macronutrient composition and obesity in youth is complex and context-dependent, highlighting the need for age and sex-specific considerations in future research and dietary guidance.

The clinical and public health implications of this study are significant. The findings caution against simplistic dietary messages that focus on restricting a single macronutrient, such as carbohydrates, for children [12]. Such an approach could inadvertently encourage the replacement of healthy, whole-food carbohydrates with unhealthy fats and proteins, potentially associated with a higher obesity risk. Instead, dietary guidance for children and adolescents should prioritize the promotion of high-quality, nutrient-dense dietary patterns. This includes emphasizing the consumption of whole fruits and vegetables, legumes, whole grains, and lean sources of protein, while limiting intake of UPFs, sugary beverages, and foods high in saturated fat [33]. The focus should be on establishing healthy, sustainable eating habits rather than on restrictive dieting, which carries risks of nutritional deficiencies and disordered eating.

This study has several notable strengths, including its large, nationally representative sample, which enhances the generalizability of the findings to the U.S. pediatric population. The use of standardized data collection protocols by NHANES and the application of sophisticated statistical methods, such as restricted cubic splines, to model complex relationships are also significant strengths. Nevertheless, several limitations must be acknowledged. First, the cross-sectional design precludes any inference of causality; it is impossible to determine whether the observed dietary pattern precedes the development of obesity or is a consequence of it. Second, dietary data were collected via 24-hour recalls, which are susceptible to recall bias and may not fully capture an individual’s long-term habitual diet. Third, the LCD score is a composite measure that does not differentiate between the quality of macronutrient sources (e.g., plant-based vs. animal-based proteins and fats), a factor known to have different health implications. Additionally, the LCD score applies equal weights to each macronutrient sub-score, which may not accurately reflect their differential impacts on obesity risk, although our sensitivity analyses suggested fat and protein were primary drivers. Finally, despite adjusting for several key confounders, the potential for residual confounding from unmeasured variables remains. Notably, a high proportion of missing data prevented the inclusion of physical activity and sleep duration in our models, which are critical pediatric determinants of obesity.

Future research should aim to address these limitations. Longitudinal cohort studies are needed to establish the temporal sequence between the adoption of specific dietary patterns and the development of obesity in children. Research using more refined dietary scoring systems that consider both food quality and processing levels, such as distinguishing between healthy plant-based LCDs and less healthy animal-based LCDs, would yield more specific and actionable insights. Intervention studies designed to improve overall dietary quality, rather than simply manipulating macronutrient ratios, are also warranted to confirm the most effective strategies for childhood obesity prevention and management.

## Conclusion

This study demonstrates a positive association between a higher low-carbohydrate diet score and the prevalence of obesity among children and adolescents. This relationship suggests that, in the general pediatric population, a dietary pattern that is de facto lower in carbohydrates is likely indicative of poor overall dietary quality and is associated with adverse weight outcomes. These findings underscore the importance of public health and clinical strategies that move beyond a focus on single macronutrients and instead promote balanced, high-quality dietary patterns rich in whole, nutrient-dense foods as a cornerstone for preventing childhood obesity.

## Supplementary Information

Supplementary Material 1.

## Data Availability

The datasets generated and/or analyzed during the current study are publicly available on the National Health and Nutrition Examination Survey (NHANES) website, [https://www.cdc.gov/nchs/nhanes/index.htm].

## Abbreviations

LCD: Low-carbohydrate diets
NHANES: National Health and Nutrition Examination Survey
BMI: Body mass index
ORs: Odds ratios
CIs: Confidence intervals
RCS: Restricted cubic splines
PIR: Poverty Income Ratio
SD: Standard deviation
UPFs: Ultra-processed foods
