# Supplementary material for: Association between low-carbohydrate diet score and childhood obesity: a national population-based study
Source: BMC Pediatr. 2026 Apr 11;26:473. doi: 10.1186/s12887-026-06835-1 (PMC13195906; doi:10.1186/s12887-026-06835-1)
Supplement: Supplementary file 1 — Supplementary Material 1. [file 12887_2026_6835_MOESM1_ESM.docx]

**Table S1**. The criteria for determining the LCD score.

| **Points** | **Carbohydrate relative intake, %** | **Protein relative intake, %** | **Fat relative intake, %** |
| --- | --- | --- | --- |
| **0** | >61.6 | <10.4 | <25.3 |
| **1** | 58.7-61.6 | 10.4-11.4 | 25.3-27.7 |
| **2** | 56.4-58.6 | 11.5-12.3 | 27.8-29.5 |
| **3** | 54.7-56.4 | 12.4-13 | 29.6-31 |
| **4** | 53.2-54.7 | 13.1-13.7 | 31.1-32.3 |
| **5** | 51.8-53.2 | 13.8-14.4 | 32.4-33.5 |
| **6** | 50.2-51.8 | 14.5-15.1 | 33.6-34.9 |
| **7** | 48.5-50.2 | 15.2-16 | 35-36.4 |
| **8** | 46.2-48.5 | 16.1-17.1 | 36.5-38.2 |
| **9** | 43.1-46.2 | 17.2-19 | 38.3-40.7 |
| **10** | <43.2 | >19.1 | >40.8 |

Note: LCD score is the sum of the scores for the three nutrients, ranging from 0 to 30.

**Table S2.** Proportion of missing data for selected covariates.

| **Variables** | **Missing count** | **Missing percentage (%)** |
| --- | --- | --- |
| **Alcohol consumption** | 20862 | 100 |
| **Smoking status** | 20862 | 100 |
| **Marital status** | 18897 | 90.58 |
| **Sleep duration (hours/day)** | 18666 | 89.47 |
| **LDL cholesterol (mmol/L)** | 16997 | 81.47 |
| **Bone mineral density** | 15472 | 74.16 |
| **Moderate physical activity** | 13769 | 66 |
| **Triglycerides (mmol/L)** | 13615 | 65.26 |
| **Sedentary time (hours/day)** | 7581 | 36.34 |
| **HDL cholesterol (mmol/L)** | 7024 | 33.67 |
| **Education level** | 5286 | 25.34 |
| **Parental education level** | 2712 | 13 |
| **Poverty income ratio** | 1316 | 6.31 |
| **Waist circumference (cm)** | 540 | 2.59 |

**Table S3.** Collinearity diagnostics (Variance Inflation Factors) for the fully adjusted multivariable logistic regression model.

| **Variables** | **GVIF** | **Df** | **GVIF^(1/(2*Df))** |
| --- | --- | --- | --- |
| **LCD SCORE** | 1.210656 | 1 | 1.100298 |
| **Age** | 1.500498 | 1 | 1.224948 |
| **Sex** | 1.056362 | 1 | 1.027795 |
| **Race** | 1.228603 | 4 | 1.026069 |
| **Poverty income ratio** | 1.23478 | 1 | 1.111207 |
| **Total energy intake** | 1.278085 | 1 | 1.130524 |
| **Parental education level** | 1.269383 | 1 | 1.126669 |
| **Education level** | 1.481953 | 1 | 1.217355 |
